# Farm Owners and Workers as Key Informants in User-Centered Occupational Health Prototype Development: A Stakeholder-Engaged Project

**DOI:** 10.2196/jmir.9711

**Published:** 2019-01-29

**Authors:** Bryan Weichelt, Casper Bendixsen, Matthew Keifer

**Affiliations:** 1 Marshfield Clinic Research Institute National Farm Medicine Center Marshfield, WI United States; 2 University of Washington and Puget Sound Veterans Administration Seattle, WA United States

**Keywords:** agriculture, farmworkers, injuries, occupational medicine, return to work, software application

## Abstract

**Background:**

The cost of workplace injuries and illnesses significantly impacts the overall cost of health care and is a significant annual economic burden in the United States. Many dairy and pork farm owners in the Upper Midwest have expanded operations and taken on the role of manager and employer yet receive little training in injury prevention, farm safety, or workers’ compensation programs and processes. Clinicians play a key role in the return to work of injured and ill farmers and farmworkers to their jobs, though little to no formal training is offered by medical schools.

**Objective:**

This stakeholder-engaged project aimed to develop a prototype application designed to assist clinicians in returning injured farmworkers to light-duty job assignments with their current employers and to assess farm owners’ and managers’ attitudes toward and barriers to adopting mobile health tools for themselves or their employees.

**Methods:**

We conducted 12 semistructured interviews with English-speaking farm owners and farmworkers from the Upper Midwest: 5 English-speaking and Spanish-speaking farmworker focus groups and 8 postproject interviews with farm owners that focused on attitudes and barriers to adoption of the developed software. Interviews and focus groups were audio recorded, and data were analyzed and thematically coded using audio coding.

**Results:**

Interviews and worker focus groups guided an iterative design and development cycle, which informed workflow design, button placement, and output sheets that offer specific light-duty farm work recommendations for the injured worker to discuss with his or her employer.

**Conclusions:**

The development of a complex prototype intended to impact patient care is a significant undertaking. Reinventing a paper-based process that can eventually integrate with an electronic health record or a private company’s human resources system requires substantial stakeholder input from each facet including patients, employers, and clinical care teams. The prototype is available for testing, but further research is needed in the form of clinical trials to assess the effectiveness of the process and the software’s impact on patients and employers.

## Introduction

The cost of workplace injury and illness represents a significant factor in the overall cost of health care and the cost of doing business in the United States. Work-related injuries account for approximately 30% of the total injury burden in the United States among working-age people [1]. Nationally, Leigh and colleagues estimated that total direct and indirect costs for work-related injury and illness were US $1,555.5 billion [2]. Workers and their families bore the highest percentage of that cost. Indirect costs at an estimated US $103.7 billion result from a combination of loss of wages and fringe benefit costs, loss of home productivity, and slowed or stopped production due to a key missing employee or replacement [2].

Accurate, up-to-date workplace injury estimates would be difficult to develop from administrative data alone, as the majority of agricultural operations do not contribute information to the 2 common sources of injury and illness data: Occupational Safety and Health Administration 300 reporting [3] and worker compensation systems, which vary greatly across US states. Most agricultural operations have fewer than 11 employees and, thus, are not required to file Occupational Safety and Health Administration 300 forms, which are the source of the Bureau of Labor Statistics data. Also, in many states, worker compensation rules exempt all but the largest agricultural employers from purchasing worker compensation [4]. Based on studies previously done in the Midwest [5,6] and research from work done on dairy and pork producers in other geographic areas [7-10], it is clear that these 2 industries endure significant injury and illness among their respective workforces.

The growth of concentrated animal feeding operations has been a reality in modern agriculture for the past 2 decades [11]. As family farms expand or fold, more agricultural operations are hiring workers who face the risks inherent in the agricultural workplace [12]. In Wisconsin and elsewhere, this rural workforce has rapidly diversified to include more immigrant and Hispanic workers, primarily from Mexico [13,14]. It is estimated that approximately 62% of milk produced in the United States is produced using immigrant labor [15]. Furthermore, the undocumented status of immigrant workers is, in itself, an occupational hazard [16]. This is further exacerbated by the already hazardous work environment, which remains among the most dangerous US industries. For example, 2015 data from the National Institute for Occupational Safety and Health shows that young workers (age<18 years) in agriculture were 44 times more likely to die on the job when compared to all other industries combined [17].

As health care expands and health insurance becomes available to more people through the Affordable Care Act [18], agricultural injuries to workers as well as farm owners will increasingly be cared for and managed by primary care practitioners (nurse practitioners, primary care physicians, and physician’s assistants) who must manage returning these workers to the workplace. However, despite the frequency with which clinicians are faced with managing the return to work (RTW) process, most have little training in the skill [19]. There also exist barriers even for those who are competent in this skill. Guzman et al [20] found that Canadian physicians perceived the willingness of the workplace to accommodate the injured worker as being the second most important factor in facilitating RTW after patient pain perception.

Programs that facilitate the early and safe return of recovering workers to some level of function in the workplace may substantially reduce employer costs and also be of significant benefit to the worker [21]. There are many factors that influence the success of early RTW efforts [22]. Among these are the perceived self-worth of the worker [23], the worker’s preinjury job satisfaction, pain severity, worker age, clinician expectations of RTW, employer attitudes, and competency of the RTW coordinator, who is typically a clinical staff member assisting with the process and serving as the linchpin between the patient’s physician and the employer [24,25]. Some of the most important factors relate to assuring that the accommodation of returning workers is efficient and effective, and legal and privacy constraints are recognized and mitigated [21,26].

In certain professions, the variability of work tasks is fewer than others. Within agriculture, despite the differences in processes and technology, the raising of large animals (pigs and cows) includes several necessary activities that are indispensable (feeding, manure management, milking, transport, and animal health management). The physical variability of tasks among these activities is relatively limited. Thus, it was feasible to develop a robust and versatile compendium of ergonomically characterized tasks in large animal agriculture to guide clinicians in returning injured workers through transitional work to a full recovery.

The RTW process may involve several key players. A clinician will generally manage the worker medically. A physical therapist, occupational therapist, or specially trained RTW professional may oversee the actual RTW (uncommon with small employers and rural practices), and the employer will interpret RTW restrictions from the clinician. RTW limitations are conveyed to the employer in a generic, simple list of the patient’s physical limitations. This approach is correct in concept but flawed in design, at least in the RTW of agricultural workers. In concept, work involves a limited number of functions: lifting, bending, standing, pulling, pushing, etc. While clinicians may be able to estimate a worker’s limitations and describe those on an RTW guidance sheet, they generally know little about what the workplace tasks truly involve, and most employers are not trained to interpret these abstract documents in the context of the workplace.

At the onset of this project, no known products existed privately or commercially that specifically addressed the RTW facilitation for agricultural employment. This project was designed to replace this speculative, abstract document with a concrete directive through a task-based software application fed from a database of occupational work task ergonomics from dairy and pork work. The system was expected to both facilitate an employer’s ability to adapt it to their own worksite and educate the clinician about the worksite. Additional benefits may include enhanced worker understanding and participation in the RTW decision making and improved communication between employer and clinician.

## Methods

### Project Aims

The primary goal of this 5-year project was to develop a compendium of tasks in the dairy and pork industries that encompasses the various types of production work and to redistribute task information through a software application to assist clinicians and employers in RTW processes for injured workers. First, we conducted key informant interviews with experts in dairy and pork production to identify the main work processes and specific work tasks that make up these processes. Second, we conducted a series of farmworker focus groups in parallel with farm owner or manager interviews, primarily to usability test the initial mock-up designs of the new RTW system interface. Lastly, we conducted follow-up interviews with farm owners or managers regarding their use of technologies, including mobile health-related tools for themselves or to manage the health and safety of their employees.

Through the first 2 years of this project, information on the tasks that make up work in dairy and pork production was collected. In a series of in-office meetings, we shared initial findings with research staff about the different processes that make up dairy and pork production and the different injury issues faced by the workforce. This information was obtained through the guidance and assistance of project advisors from the Professional Dairy Producers of Wisconsin, the largest dairy organization in Wisconsin; the Pork Board; and others who were interviewed during the coordinated key informant process (year 1) described below. This process also educated our team occupational therapists, who were tasked with collecting ergonomic data (years 1-2), about the terminology necessary for effective communication with dairy and pork producers.

### Coordinated Key Informant Interview Process

There were 3 separately funded occupational health research projects running in parallel that all employed key informant interviews at the start of their project activities. The 5-year projects coordinated their interviews to allow an economy of scale in these data collection processes, saving funds and minimizing demands on busy advisees and respondents. Each of the 3 projects identified key informants individually. Project personnel identified gaps in the list and suggested additional informants to be interviewed. There were 6 farm owners with employees who participated in these initial interviews. Of these participants, 3 were male and 3 were female. Questions were developed, pilot tested, and reviewed by each of the 3 project teams based on their projects’ information needs. This allowed the assembly of questions tailored to the key informant being interviewed and inclusive of the questions that each of the projects wanted to be addressed, while not overburdening participants with multiple interviews.

### Ergonomic Data Collection

The project team employed a modified version of DSI Work Solutions, Inc.’s unpublished methodology for collecting ergonomic measurements of the functional job assessments of work tasks. The codeveloper of the decision support initiative (DSI) methodology (and consultant to the project) worked with the authors to develop a nonproprietary tool that can remain for use in the public domain to collect information on functional job assessments (or analysis) in pork and dairy production. This tool was designed to allow ease in transferring data to the RTW guidance software application database. Functional job assessments or job demand analyses exist for many jobs in nonagricultural settings. This project took steps to develop these profiles for common tasks in dairy and pork which are now available within the prototype app. Lessons learned from the collection and integration of ergonomic data into a software application will be discussed in another manuscript.

### Focus Groups

Farmworkers were recruited for focus groups from among Anglo and Hispanic workers through our participant dairy and pork workplaces in Wisconsin. These farmworkers were asked to review and interpret RTW instructions. English worker instructions were translated into Spanish by a bilingual research specialist and reviewed by Marshfield Clinic Health System (MCHS) interpreters.

Instructions for RTW specialists were tested with occupational medicine providers at MCHS. Clinicians at MCHS in primary care and occupational medicine were recruited to review and offer advice on the forms designed for guiding clinicians in returning a worker to light-duty job activity. The results from the clinician interviews will be described in another paper.

The objectives of the focus groups and interviews were 3-fold: (1) to assess participants’ perception of the current process of returning an injured farmworker back to work, potentially in a limited capacity, and to see where improvements could be made; (2) to gauge the acceptance of the concept of suggesting potential tasks along with the physical limitations and treatment instructions normally included on the worker’s compensation report; and (3) to capture these data from farm employers and their employees, specifically including data from the Spanish-speaking workers—a vulnerable, yet growing population of workers in the Upper Midwest dairy industry. Feedback from the focus groups and interviews were used to help steer the initial designs and the iterative development of the software and its outputs. Focus groups have shown to open up discussion and conversation, more so than just one-on-one interviews, particularly in software design but also among Spanish-speaking Wisconsin dairy workers [27,28].

**Figure 1:**
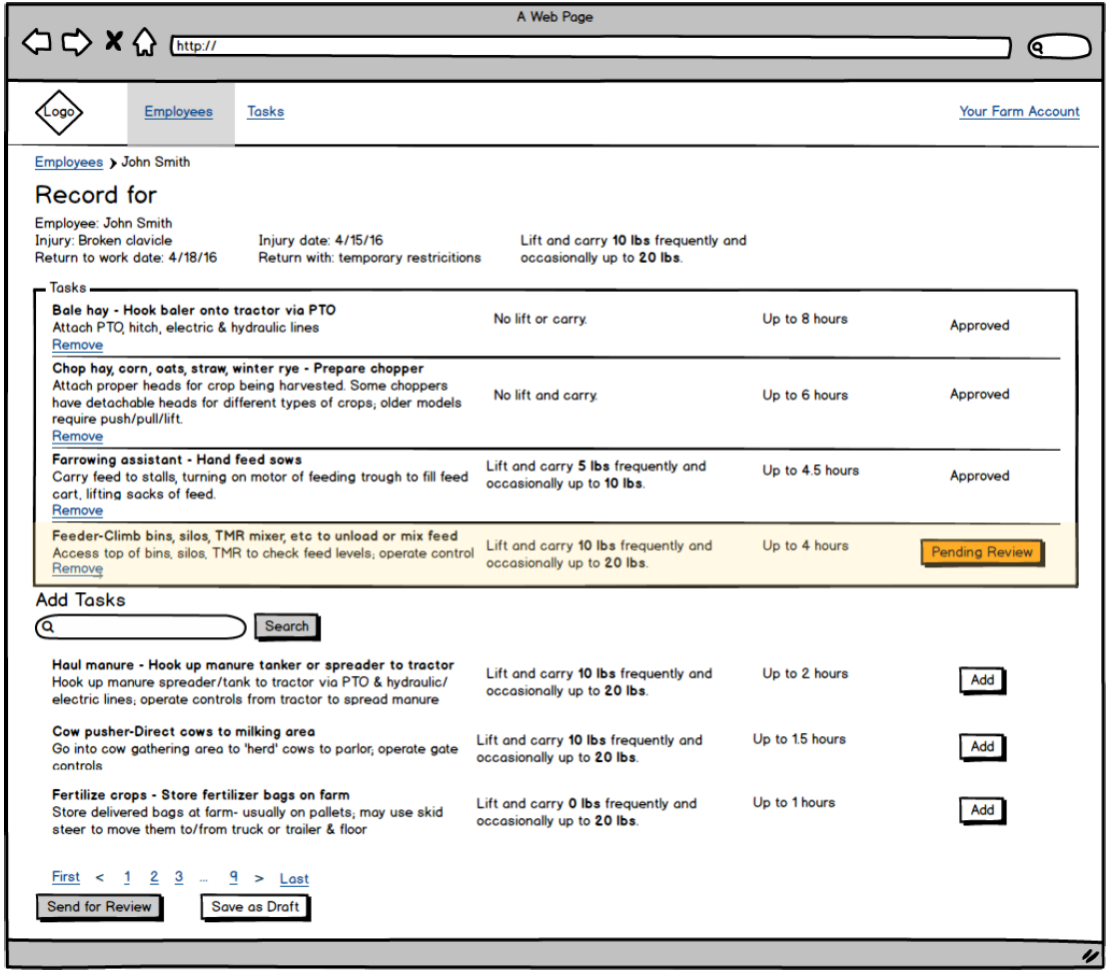
Early mock-up of the application interface.

We conducted 5 farmworker focus groups on Wisconsin dairy farms; 3 were Spanish-speaking, and 2 were English-speaking groups. The focus groups took place over the participants’ lunch hour or at a time their schedule allowed. Participants were provided with a box lunch and also received a US $20 gift card. A usability analyst facilitated the focus groups in a private area without the employer or manager present. An interpreter was present for the Spanish-speaking groups to translate all questions and answers for the benefit of the usability analyst and the participants. The usability analyst presented the existing paper-based workers’ compensation form used in clinical practice at the MCHS and facilitated a semistructured discussion regarding participants’ experience with existing RTW processes. The analyst also introduced the concept of a software application that would utilize an algorithm to present potential work tasks based on the physical limitations presented by the injury, asking questions regarding the design, button placement, and terminology. Paper copies of early design mockups of the application interface were handed out and referred to during the process (Figure 1).

Participants in each of the 5 focus groups were asked a series of questions from a semistructured questionnaire; these questions focused on elements of the Workers’ Compensation process including past injuries, experiences, and feedback on the detailed variables of the new forms’ mock-up designs.

### Interviews

**Figure 2:**
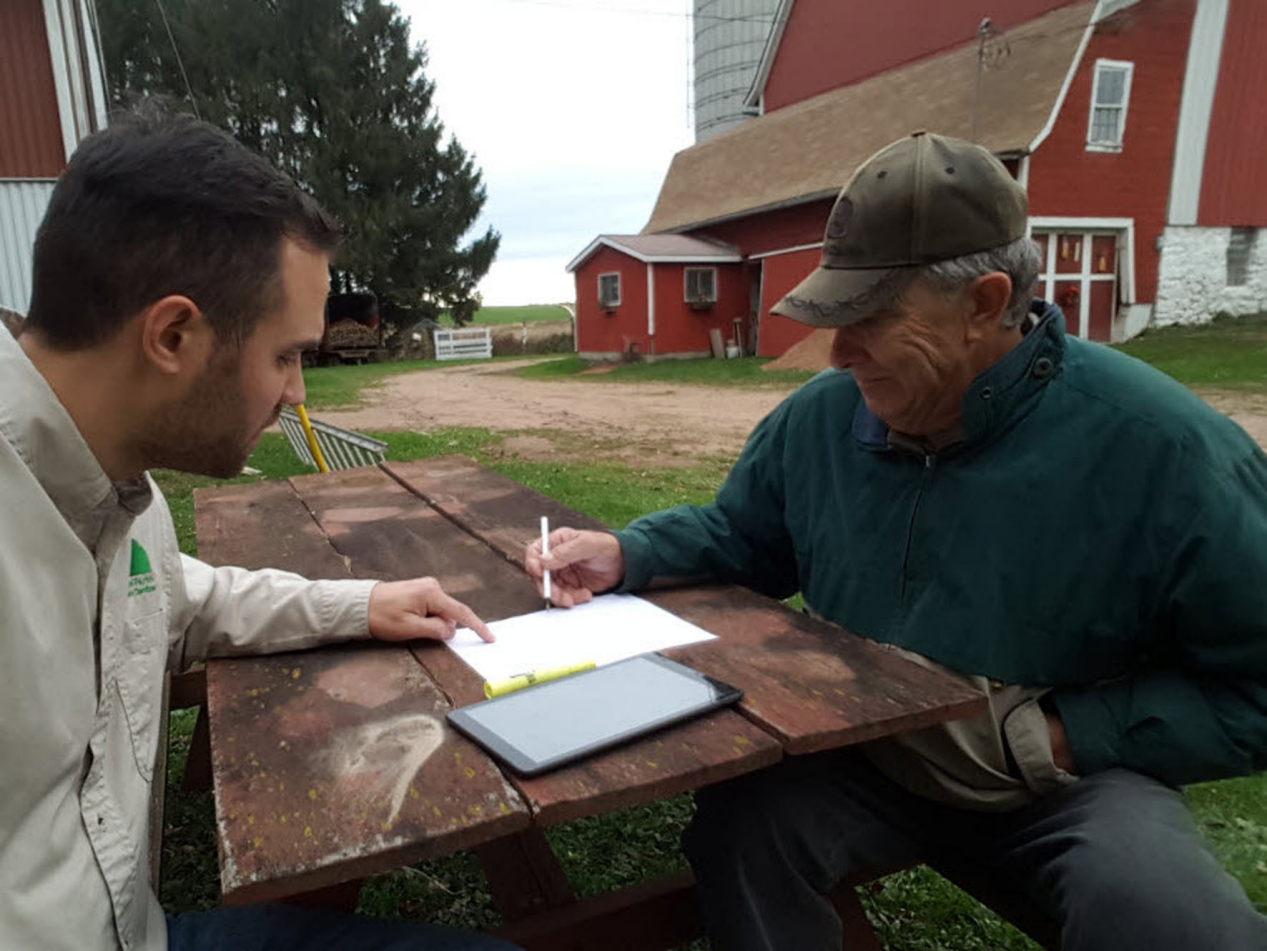
Photo of an interview with a farm owner or manager using the SafeReturnToWork.org application.

We conducted 6 interviews with Wisconsin dairy farm owners or managers to assess the current practices and level of knowledge and to gather insight on initial conceptual designs of a proposed output sheet from the SafeReturnToWork.org (SRTW) app. In total, 2 participants who were owners and 4 were farm managers or identified as administrators; 3 were male and 3 female. All 6 farms employed nonfamily full-time workers. The workers’ compensation form was a standard form that MCHS was using at the time of this study. The initial conceptual design was referred to and guided the semistructured farmer interviews (see Figure 2).

### Analysis

Interviews and focus groups were audio recorded. The primary method of analysis was audio coding [29]. The analyst and authors used inductive analyses to identify themes and organize them into thematic sections to inform the iterative software development cycle, as highlighted by participants’ direct quotes in the results section below.

## Results

### Participants’ Experience with the Workers’ Compensation Process and Claims

A substantial majority of the participants had some knowledge and experience regarding the workers’ compensation process. There was 1 farmer who was knowledgeable about the costs associated with an injury claim and spoke specifically about the multiplier effect of accepting wages. The participant stated the following:

the wages can kill you. You can have an awful lot of medical expenses, but compared to wages...We would have been way better off on the experience mod, if we did not take any wages

You know with all [name omitted]’s hospitalization, and all the surgeries on his foot and the physical therapy, uhm, and the minimal amount of wages that we took, you know, someone estimated to me that the wages probably were ten to twenty-fold impact. It was unbelievable. When they do your whole mod, this long long calculation, wages apparently are weighted, in those calculations, significantly more than medical. And I just had another person tell me too, as he was showing me their lost claims on workers’ comp, but they had a way lower mod than us, and he said’ because he’s really trying not to have lost wages. He said if you can find something for them to do, you’re way better off than taking the lost wages.

Other participants discussed their experience in managing worker claims by discussing complexities, challenges, and the accommodations made. There was 1 farmer who noted specific challenges, namely variation in available work options due to seasonality. The dairy industry is a year-round operation. Thus, many workers are immigrants, not migrant workers, meaning they have immigrated (moved permanently) to the area rather than visiting for temporary employment. This is a result of the demand for steady, year-round labor. Cows on dairy operations are milked 2 to 3 times per day, every day of the year. Some operations only shut down long enough to clean and wash the area, then start milking another section of the herd. Even with the year-round labor demand, there is still some fluctuation in the farm tasks. Spring (tillage and planting), summer (hay harvest), and fall (grains harvest) are still the busiest seasons in terms of the hours of labor needed to operate a successful dairy. Thus, winter has a lull in available light-duty job assemblies for injured workers. A participant mentioned the following:

In the summer we’re a little more flexible, but in the winter we don’t have that flexibility. So that was our one big one, and it was a winter injury, we just didn’t have the work that was matching, so. The summer is much easier for us, I’m sure it depends on each business.

Another farmer less familiar with the process noted that their operation just does not have as much flexibility in positions. He stated the following:

The conundrum is that we don’t really have any work that’s sedentary. The very light work is very minimal. You know, carrying ten pounds, not bending over, that kind of thing, we just don’t have lot of that work. Even light work, you know it’s, they’re lifting their hands above their waste, they’re reaching, all those kinds of things put them in those categories where they can’t really do a whole lot for us

When discussing the topics of seeking care and the number of claims filed, a discussion with 1 farmer led into the mix of culturally-based decision making in regard to the appropriateness of seeking care for subjectively minor injuries. During an interview with a farmer who employs English-speaking and Spanish-speaking workers on the dairy operation, 1 incident, in particular, was highlighted. The participant noted that all the claims the farm has had have come from English-speaking workers and went on to say the following:

The Hispanics, uh, they uh, they’re pretty careful about that stuff. I mean ah going to the doctor if they’re injured on the job. They’re real fix-it-myself. We had one guy that cut his leg, and uhm didn’t want anyone to know, one of the employees told me, so I went over to talk with him. and I said you cut your leg, he said yup, I know how to take care of my leg, I know that when I get home tonight I’m going to elevate it. I don’t need stitches. I said I need to see it and he showed it to me and it didn’t need stitches. He said I know how to clean a wound, I know how to take care of it, I don’t need to go to a doctor. Ok. You know, it wasn’t one that needed stitches. If it needed stitches it would be a little different story. But they’re pretty careful about that stuff.

### Initial Conceptual Designs of the SafeReturnToWork Software Interface

There was a mix of responses among farm owner or manager interview participants when reviewing the conceptual designs. This was also the most involved and time-consuming section of the interview, where participants delved into specific sections of the output sheet (eg, physician contact information, checkboxes vs narratives, suggested tasks, limitations, medications, and treatment plan).

When reviewing the section regarding physician contact information, 1 participant was very quick to respond. Its usefulness was questioned immediately, based on the experience of this farmer and the perception of the transpired events. The interviewee stated the following:

I do not think a doctor will talk to us. I don’t think it matters. HIPAA. They won’t talk, I can’t call and ask about my children that are 21 years old, why would they allow me to call and talk to them about an injured farm worker?

We haven’t had, I can think of two of them, where we tried, and we didn’t get. Maybe it would be just the contentious nature of their injury and those two individuals were you know, I don’t know. but I did not, those two we did try and we just decided that we cannot do that. So we just went back to our insurance company and had them do what they can on that end to try and get somewhere.

Interviewer: So when you tried to call the doc, did they refuse to speak with you?

yea, they wouldn’t call back”

Finally, farm owners or managers were asked if they thought Web-based resources would be useful for an injured farmworker and if they would access those resources. When asked about thoughts on using the internet to access therapy- and recovery-related resources to aid in the rehabilitation process of an injured patient, one farmer responded by stating the following:

For me, that would not be customer service at all. Give them the information in hard copy, I think is the customer service route. Because I don’t think if I’m partially laid up, and I’ve been injured at work, that I should have to come home, search the internet, find the website, find the information, print it out. I’m not going to do the work. If it’s sitting around on the counter, my kids or my spouse will probably say, hey your doctor gave you this, you better get going on this. If I have to go to a computer and find it, I don’t think it’s ever gonna get looked at, we’re done

During these farm owner or manager interviews, further input was offered on a number of related topics, including some foresight into the usefulness of a system to inform future health and safety interventions. There was 1 such farmer who was specifically interested in the outcomes of the Safe RTW project in tracking data and advising the industry on best practices that will lead to fewer injuries, fewer claims, and cost savings.

I see the end result to say, workers comp rates on dairy farms – here are the injures – here are the bulk of injuries that farms have with workers comp. What can we do as an industry to minimize these high number injuries, from a client perspective, to bring down our work comp rates? That’s the other side of it, as an industry, what can we gain by those types of things, by knowing those types of things. Because in five years if you guys go back to your data warehouse and say the number one workers comp [claim] is eye injuries or wrist injuries…how do these accidents happen and what causes those injuries. You guys as dairy farmers, if you eliminate these situations how much less work comp losses would you have on your bottom line?

### Focus Groups with Farmworkers

A total of 35 farmworkers participated in the 5 focus groups comprising 3 Spanish-speaking and 2 English-speaking groups (see Table 1). A total of 32 males and 3 females participated. Of the farmworkers, 20 identified as Hispanic/Latino, 14 identified as white or Caucasian, and 1 did not self-identify. Spanish was primarily spoken by 20 workers. The project team did not collect any further demographic data from these participants. However, it is likely that the Spanish-speaking participants had similar home states, limited prior agricultural work experience, and similar current jobs in dairy (milkers, pushers, and feeders) as reported by Liebman et al [16].

These focus groups produced significant design changes to the prototype light-duty job activity forms, which shifted to a design that closely resembles the current workers’ compensation form used throughout the MCHS.

### SafeReturnToWork Adoption

On-farm interviews were conducted with farm employers, inquiring about the possible use of this technology and the concept of engaging with physicians about workers’ compensation related issues. In the final year of this project, 8 farmers were interviewed to further explore barriers to adoption of the SRTW system and its mobile format in regard to the use of mobile health-related tools. Interviewees included married couples, single farmers, and farm managers. All participants had previously engaged with research staff regarding the SRTW system at different points in its development. The interviews were specifically focused on how farmers have communicated with physicians regarding injured employees in the past and how the SRTW system could aid and improve that communication. Discussions gravitated toward the strengths and weaknesses of the paperwork an injured worker would return with from the physician, the attractiveness of having the same information over the Web in a more useful format, and how farmers and farm managers would prefer to communicate during a light-duty work period. Interview and data analyses were conducted using the same methods noted in this paper.

Sharing a specific story, 1 farmer articulated a common theme regarding the difficulties of communicating what a light-duty regimen could consist of with a physician and why it is necessary to the worker. This particular worker was injured by a dairy cow and sought medical care for a broken rib and punctured lung. The worker was assigned a 45-day light-duty requirement from the physician. However, in as early as 20 days, the worker was asking to come back to work, afraid of either being fired or fearing there would be repercussions regarding his work in the future, despite the employer’s reassurance. Similar worker fears were uncovered through focus groups by Liebman and colleagues [16]. The farmer felt the physician could have aided him more in explaining why a light-duty regimen was necessary. The farmer reported 2 written communications from the physician, both of which he felt were difficult to understand and were uninterpretable by the worker, so he opted for a more restrictive regimen. For example, while work in a skid steer would have suited the employee’s skills, experience, and the light-duty work orders, the farmer felt that the climbing in and out of the skid steer was beyond the recommendations. He agreed with the interviewers that he would have utilized the proposed version of the SRTW system to find more jobs for the worker and to further explain the possible tasks to the physician.

Another farm couple with a similar story stated the following:

We’re probably making assumptions about [the physician’s] job and we know he’s making assumptions about ours. […] It couldn’t hurt to be able to provide education both ways.

All interviewees agreed that the proposed enhancements to the system would be helpful to farms with many employees (most participants had 15 or more). They also felt that workers’ compensation insurance carriers should be considered users and financiers of such a technology, making the argument that it was their business to keep a client’s employees healthy during a light-duty assignment.

### Unexpected Outcomes and Deliverables

**Figure 3:**
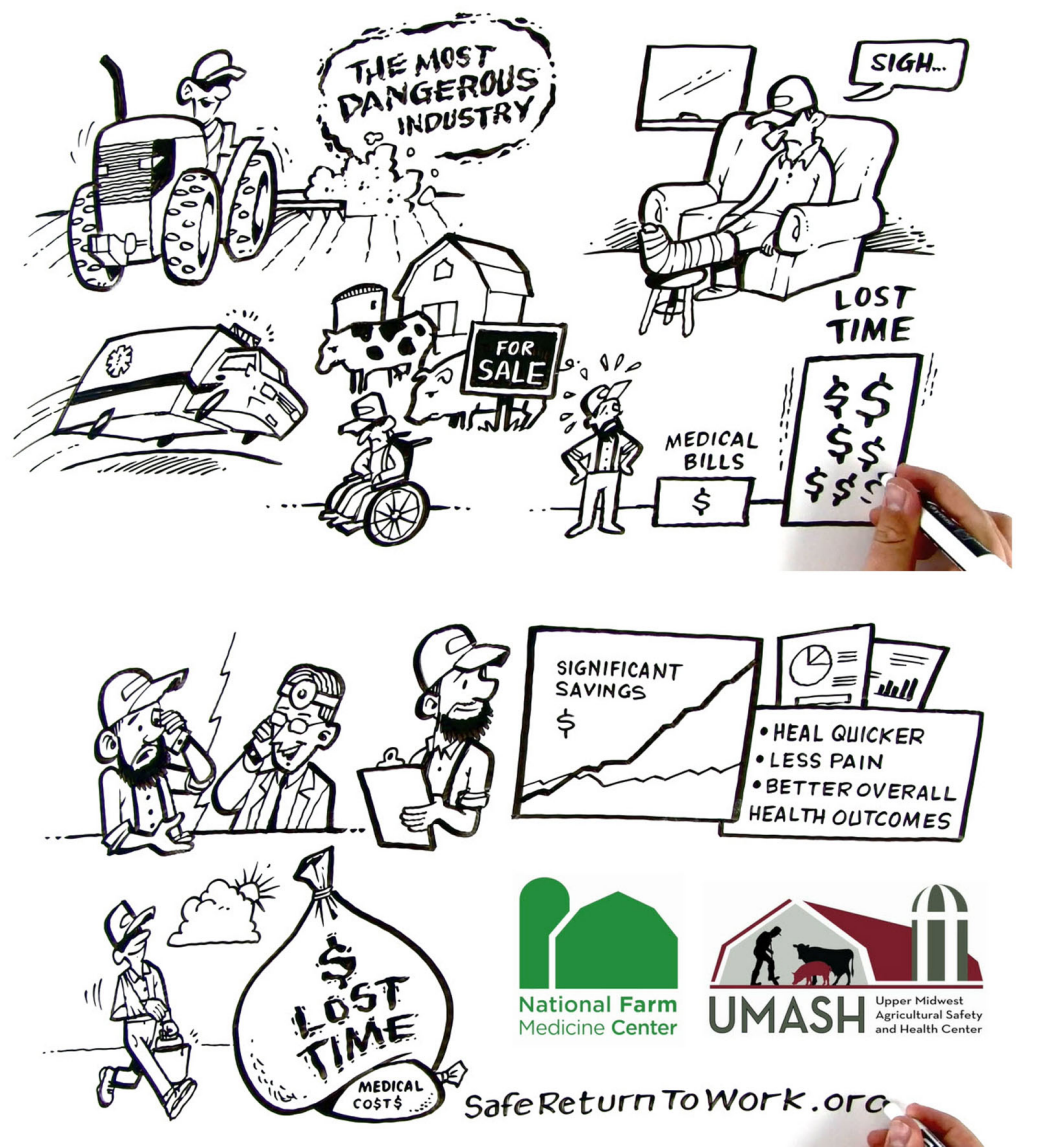
Screenshots of the 90-second whiteboard video. (Source: created by authors).

The interview findings led the research team into an unplanned investigation of workers’ compensation calculator variation across the Upper Midwest. The results of those investigations are discussed in another manuscript. The team also created an informational page on the SRTW website specifically designed to educate farm owners and managers of the costs related to workers’ compensation claims for agricultural operations. That information was reviewed by 3 representatives of workers’ compensation companies in Wisconsin and Minnesota and is now available on the calculator tab at SafeReturnToWork.org. Furthermore, a 90-second educational whiteboard video (Figure 3) was developed to assist with this same effort—educating farm owners or managers—the script was reviewed by 3 representatives of workers’ compensation insurance companies in Wisconsin and Minnesota. This Web-based video had been disseminated to farmers via email and Facebook posts by the Professional Dairy Producers of Wisconsin, an original key informant of this project.

**Table 1:** Summary of focus groups (N=35).

Focus group number	Language spoken during focus group	Males, n (%)	Females, n (%)	Location	Wisconsin region
1	Spanish	5 (14)	0 (0)	Dairy farm	Central
2	English	8 (23)	0 (0)	Dairy farm	Western
3	Spanish	7 (20)	0 (0)	Dairy farm	Western
4	English	4 (11)	2 (6)	Dairy farm	Central
5	Spanish	8 (23)	1 (3)	Dairy farm	South Central

## Discussion

### Principal Findings

The owner-operator of an agricultural operation can often describe the entirety of his business to a clinician treating him or her for an injury. The owner-operator can incorporate and test his or her own injury-imposed limitations in the process of completing the workday. However, an employee generally has less knowledge of alternative work options and does not have the autonomy to decide which job tasks to take on. The clinician must establish limitations for the injured worker that are transmitted to the workplace decision maker (owner-operator or supervisor) who interprets these limitations and decides what work is possible. All too often, the workplace decision maker, confused by the speculative limitations, declares the worker unsuitable for RTW. This was reaffirmed in the interviews with farm employers.

The availability of the prototype developed during this project may continue to open communication lines with rural practitioners. Electronically linking relevant training materials to the decisions being made during the interaction with the program will put educational content and materials at the fingertips of the user, providing clinical decision support at the point of care.

Data collection with useful and actionable clinical decision support is important, if not critical, when implementing clinical systems. Once the prototype has been further tested, we anticipate expansion of the capabilities of the program by including data-gathering capabilities, allowing clinicians to track various aspects of patients’ RTW. This could include the time from injury to various stages of work activities. In future iterations, we expect to include data capture such as demographics, injury type, and progress monitoring. These will provide patients and providers with the ability to understand the success of their RTW activities and track their progress in RTW management over time.

### Limitations

While this project collected a near complete compendium of tasks likely to be found on dairy and pork farms, there may be substantial variability among smaller farms that this project cannot accommodate. However, smaller farms employ fewer employees, and some are run solely by the owner and family members. Thus, they are less likely to fall within a formal RTW program overseen by an employer and guided by a health care team. The system does, however, cover the vast majority of tasks on large farms and information appropriate for the majority of workers in the dairy and pork industries. Furthermore, the participants of the study and the host sites used for ergonomic data collection were primarily in Wisconsin and may not be representative of dairy and pork operations outside of the Upper Midwest.

Analyzing the functional job demands is a task that will present some variability. The descriptions by one observer may differ to some degree from the descriptions of another. We used single individuals in these tasks and did not perform repeat analyses. We chose to work with occupational therapists who were trained in the DSI methodology in an attempt to limit this variability. The DSI training is thorough and consistent, and we anticipated that this approach would reduce variability.

### Conclusions

Farmers and farmworkers are increasingly adopting new technologies in their personal and professional lives, and the agricultural sector is quickly advancing in technological sophistication, from robotic milking systems to autonomous tractors and unmanned aerial vehicles. It was anticipated that farmers and farmworkers would not only be suitable as key informants, but they would also be critical in the development of an application designed to benefit the farming industry.

This translational health informatics project has produced a prototype that could be useful to rural practitioners caring for patients injured in dairy and pork production work. We believe these practitioners, the workers they care for as patients, and the employers will benefit from the guidance provided by the program. Further development of the RTW system will be pursued with subsequent funding. A future line of research may include clinical trials of the program in Wisconsin and Minnesota comparing the case parameters (eg, duration of time loss, duration of light-duty, and duration until return to full duty) of the program to statistics collected using standard practice in returning workers to work in the dairy and pork industries.

Since there is a significant financial incentive to return injured workers to a light-duty job, limiting time loss, it is unlikely that clinicians would face barriers among farmers in adopting RTW technology. However, there is little incentive on the part of clinicians to adopt the technology as is. Without seamless integration into the clinical electronic health record workflows, it is unlikely that individual physicians would consistently leverage a system such as this at the point of care without external influence or incentive. Future research should also focus on bridging gaps that appear to exist between workers’ compensation insurers and physicians in the RTW process.

## Abbreviations

DSI: Decision support initiative
HIPAA: Health Insurance Portability and Accountability Act of 1996
MCHS: Marshfield Clinic Health System
RTW: return to work
SRTW: SafeReturnToWork.org

## References

[1] Smith GS, Wellman HM, Sorock GS, Warner M, Courtney TK, Pransky GS, Fingerhut LA (2005). Injuries at work in the US adult population: contributions to the total injury burden. Am J Public Health.

[2] Leigh JP, Markowitz SB, Fahs M, Shin C, Landrigan PJ (1997). Occupational injury and illness in the United States. Estimates of costs, morbidity, and mortality. Arch Intern Med.

[3] US Department of Labor Occupational Safety and Health Administration.

[4] Kirkhorn SR, Earle-Richardson G, Banks RJ (2010). Ergonomic risks and musculoskeletal disorders in production agriculture: recommendations for effective research to practice. Journal of Agromedicine.

[5] Nordstrom DL, Layde PM, Olson KA, Stueland D, Brand L, Follen MA (1995). Incidence of farm-work-related acute injury in a defined population. Am J Ind Med.

[6] Gerberich SG, Church TR, McGovern PM, Hansen HE, Nachreiner NM, Geisser MS, Ryan AD, Mongin SJ, Watt GD (2004). An epidemiological study of the magnitude and consequences of work related violence: the Minnesota Nurses' Study. Occup Environ Med.

[7] Rautiainen RH, Reynolds SJ (2002). Mortality and morbidity in agriculture in the United States. J Agric Saf Health.

[8] Waller JA (1992). Injuries to farmers and farm families in a dairy state. J Occup Med.

[9] Pratt DS, Marvel LH, Darrow D, Stallones L, May JJ, Jenkins P (1992). The dangers of dairy farming: the injury experience of 600 workers followed for two years. Am J Ind Med.

[10] Douphrate DI, Rosecrance JC, Wahl G (2006). Workers' compensation experience of Colorado agriculture workers, 2000-2004. Am J Ind Med.

[11] Gurian-Sherman D (2008). Union of Concerned Scientist.

[12] Jesse E (2008). MARKETING AND POLICY BRIEFING PAPER.

[13] Harrison J, Lloyd S, O'Kane T University of Wisconsin.

[14] Thilmany D Colorado State University.

[15] Rosson P, Adcock F, Susanto D, Anderson D (2009). The economic impacts of immigration on U.S. dairy farms.

[16] Liebman A, Juarez-Carrillo Patricia Margarita, Reyes Iris Anne Cruz, Keifer Matthew Charles (2016). Immigrant dairy workers' perceptions of health and safety on the farm in America's Heartland. Am J Ind Med.

[17] NIOSH (2018). Analysis of the Bureau of Labor Statistics Census of Fatal Occupational Injuries microdata. Morgantown, WV: U.S. Department of Health and Human Services, Centers for Disease Control and Prevention, National Institute for Occupational Safety and Health.

[18] Patient ProtectionAffordable Health Care Act (2010). One Hundred Eleventh Congress of the United States of America, the Second Session.

[19] Pransky G, Katz JN, Benjamin K, Himmelstein J (2002). Improving the physician role in evaluating work ability and managing disability: a survey of primary care practitioners. Disabil Rehabil.

[20] Guzman J, Yassi A, Cooper JE, Khokhar J (2002). Return to work after occupational injury. Family physicians' perspectives on soft-tissue injuries. Can Fam Physician.

[21] Special Committee on Health‚ Productivity‚Disability Management‚ American College of OccupationalEnvironmental Medicine (2009). Healthy workforce/healthy economy: the role of health, productivity, and disability management in addressing the nation's health care crisis: why an emphasis on the health of the workforce is vital to the health of the economy. J Occup Environ Med.

[22] Shaw WS, van der Windt Danielle A, Main CJ, Loisel P, Linton SJ (2009). Early patient screening and intervention to address individual-level occupational factors ("blue flags") in back disability. J Occup Rehabil.

[23] Shaw W, Hong Q, Pransky G, Loisel P (2008). A literature review describing the role of return-to-work coordinators in trial programs and interventions designed to prevent workplace disability. J Occup Rehabil.

[24] Shaw WS, Linton SJ, Pransky G (2006). Reducing sickness absence from work due to low back pain: how well do intervention strategies match modifiable risk factors?. J Occup Rehabil.

[25] Shaw WS, Pransky G, Patterson W, Winters T (2005). Early disability risk factors for low back pain assessed at outpatient occupational health clinics. Spine (Phila Pa 1976).

[26] Tamminga SJ, van Hezel Sanne, de Boer Angela Gem, Frings-Dresen MH (2016). Enhancing the Return to Work of Cancer Survivors: Development and Feasibility of the Nurse-Led eHealth Intervention Cancer@Work. JMIR Res Protoc.

[27] Keifer Matthew C, Reyes Iris, Liebman Amy K, Juarez-Carrillo Patricia (2014). The use of audience response system technology with limited-english-proficiency, low-literacy, and vulnerable populations. J Agromedicine.

[28] Liebman Amy King, Juarez-Carrillo Patricia Margarita, Reyes Iris Anne Cruz, Keifer Matthew Charles (2016). Immigrant dairy workers' perceptions of health and safety on the farm in America's Heartland. Am J Ind Med.

[29] Wainwright M, Russell A social research Update.

